# Diagnosis and therapeutic outcomes of biopsy-confirmed Pure Neuritic Leprosy: A two-decade experience

**DOI:** 10.1371/journal.pntd.0014212

**Published:** 2026-04-10

**Authors:** Shrimantika Sarkar, Angel Miraclin T, Ranjani Jayachandran, Anitha Jasper, Reethu Anny Eapen, Lydia Mathew, Dharshini Sathishkumar, Dincy Peter, Leni George, Harshad Arvind Vanjare, Deepti Bal, Hemalatha R, Aditya V. Nair, Shaikh Atif Iqbal Ahmed, Appaswamy Thirumal Prabhakar, Vivek Mathew, Sanjith Aaron, Susanne Pulimood, Rima Sathyakumar, Geeta Chacko, Ajith Sivadasan

**Affiliations:** 1 Department of Neurological Sciences, Christian Medical College, Vellore, Tamil Nadu, India; 2 Department of General Pathology (Division of Neuropathology), Christian Medical College, Vellore, Tamil Nadu, India; 3 Department of Dermatology, Christian Medical College, Vellore, Tamil Nadu, India; 4 Department of Radiodiagnosis, Christian Medical College, Vellore, Tamil Nadu, India; Yale University School of Medicine, UNITED STATES OF AMERICA

## Abstract

**Background:**

Pure neuritic leprosy (PNL) is a distinct form of leprosy characterized by peripheral nerve involvement without skin lesions. Diagnosis is often delayed, and ‘silent neuritis’ contributes to progressive and irreversible nerve damage. Despite appropriate therapy, many patients develop long-term disability. This study describes the clinical profile, diagnostic patterns, therapeutic outcomes, and predictors of disability in biopsy-confirmed PNL.

**Methods:**

We conducted a retrospective observational cohort study at a tertiary referral centre in South India, enrolling biopsy-confirmed PNL cases from January 2003 to May 2023. Demographic, clinical, electrophysiological, and histopathological data were collected. The primary outcome was the proportion of patients with high disability at follow-up (WHO grade 2). Predictors of disability were identified using multivariate analysis.

**Results:**

Ninety-five patients were included (mean age 43.02 years (SD 15.32); 77% male). Over half (55%) presented 1–5 years after symptom onset. Sensory deficits were most common (80%), followed by foot drop (51%), ulnar claw (38%), and trophic ulcers (25%). Electrophysiology revealed mononeuritis multiplex in 52.6% and axonal polyneuropathy in 22.2%. Nerve biopsy showed borderline tuberculoid pathology in 78% and borderline lepromatous in 15.7%. Notably, 6 patients with mononeuropathy had lepromatous pathology. All patients received multidrug therapy; 41 experienced lepra reactions. Higher severity at treatment initiation was significantly associated with persistent disability.

**Conclusions:**

PNL often presents late, with many patients already experiencing nerve damage. A substantial subset of clinically mononeuritis patients demonstrated lepromatous pathology, emphasizing the need for nerve biopsy in all suspected cases. Early detection and timely treatment may reduce disability burden. These findings highlight critical gaps in current diagnostic strategies and underscore the importance of aggressive early intervention in PNL.

## Author summary

Pure neuritic leprosy (PNL) is an under-recognised but important form of leprosy in which only the nerves are affected without visible skin lesions. This leads to delayed diagnosis, often after irreversible nerve damage has occurred. Our study describes one of the largest single-centre experiences with biopsy-confirmed PNL over a two-decade period in an endemic setting. We analysed the clinical, electrophysiological, and histopathological characteristics of 95 patients to identify factors associated with long-term disability. Most patients presented years after symptom onset, and more than half already had significant nerve dysfunction. Sensory loss and motor deficits such as foot drop and ulnar claw were common. A key finding was that several patients with clinical mononeuropathy had lepromatous disease on biopsy, underscoring the diagnostic value of nerve biopsy in this group. A higher disability at the time of diagnosis was the strongest predictor of persistent disability at follow-up. These findings highlight major gaps in the early detection and management of PNL. Early clinical suspicion, timely biopsy, and prompt therapy are critical to preventing lifelong disability. Strengthening diagnostic strategies for neuritic forms of leprosy is essential to achieve the global goal of zero disability and improved quality of life for affected individuals.

## Introduction

The earliest description of PNL was by Albert Neisser in 1903, when he coined the term ‘neural type of leprosy’ or ‘lepra nervorum.’ [1]. This was further defined at the International Congress of Leprosy held in Madrid in 1953, where it was recognized as a distinct entity. The prevalence of PNL in India ranges from 4% to 18%, with higher rates in South India. [2–7]. The pathogenesis by which *Mycobacterium leprae* causes nerve damage is intriguing, as Schwann cells appear at the initial site of entry, and the infected nerves serve as the epicentre of inflammation secondary to a robust immune response that persists for days to years. [7–10]. Segmental demyelination appears to be the pathognomonic response to infection, followed by regeneration and fibrosis, which can lead to permanent disability. [7,8]. In an interesting study by Rambukkana et al. using a mouse model, the bacilli cause rapid contact-dependent demyelination, which can be evoked by dead bacteria or the bacterial cell wall.[11]. Without an immune response, an intense proliferation of non-myelinated Schwann cells occurs, which could harbour a greater bacterial load than myelinated Schwann cells. [11]. More than 50% have a high disability (WHO disability - 2) at presentation because of ‘silent neuritis’ or ‘quiet nerve paralysis,’ which is defined as a deficiency of the nerve and/or motor function without pain or sensitivity of the nerve.[2,3,12]. The occurrence of lepra reactions during therapy further leads to cumulative disability. This study describes our experience in evaluating and managing PNL, their clinical profile, electrophysiological characteristics, histology, and long-term outcomes. We also attempted to identify the predictors of severe disability.

## Materials and methods

### Ethics statement

The study was approved by the Institutional Review Board of Christian Medical College, Vellore (IRB Min No. 2507132). Since it is a retrospective study, the ethics committee waived informed consent. Written consent was obtained for the publication of patient-related MRI and histopathological images.

### Methods

This is a retrospective descriptive study from a tertiary care centre in South India, involving hospitalized patients with biopsy-confirmed PNL from January 2003 to May 2023. All patients underwent dermatological evaluation and split-skin smears at diagnosis to exclude cutaneous involvement. The demographic data and clinical findings, including nerve thickening, nerve function impairment, and disability, were documented. Electrophysiological characteristics and histopathological findings from the nerve biopsy were reviewed.

### Case definitions

1. Pure neuritic leprosy was defined as leprosy patients presenting with exclusive nerve or nerve trunk involvement without skin findings, negative slit skin smears, and diagnosis established by nerve biopsy showing features of leprous neuritis.
2. Sensory impairment: The inability or reduced ability to appreciate temperature, pain, and/or fine touch was considered a sensory function impairment.
3. Motor impairment was diagnosed when less than grade 5 power was recorded on voluntary muscle testing on the Medical Research Council scale.
4. Clinical evidence of involvement of a single nerve due to leprosy (nerve thickening associated with nerve function impairment along the nerve supply) was categorized as mononeuropathy, asymmetric involvement of multiple nerves as mononeuritis multiplex, and symmetric involvement of multiple nerves as polyneuropathy.
5. We defined high disability as a WHO disability score of 2 [13].
6. Axonal polyneuropathy: Axonal polyneuropathy was defined by reduced compound motor action potential (CMAP) and/or reduced sensory nerve action potential (SNAP) amplitudes with relatively preserved conduction velocities and absence of electrodiagnostic features of demyelination.
7. Demyelinating polyneuropathy: Demyelinating polyneuropathy was defined based on established criteria (EFNS/PNS 2021) requiring abnormalities in conduction velocity, distal latency, F wave latency, conduction block, or temporal dispersion in at least two motor nerves.

### Inclusion criteria

We included adults aged 18 or older who met the criteria for PNL.

### Outcomes

The primary outcome was the proportion with high disability, defined as WHO grade 2 at follow-up.

### Statistical analysis

Descriptive summaries were reported as mean (SD) or median (IQR) for continuous variables and frequencies/percentages for categorical variables. While the differences between the groups for continuous variables were analysed using an independent Student’s T-test and Mann-Whitney U test as applicable, differences in categorical variables were analysed using the chi-square test. All statistical tests were 2-tailed, and p-values < 0.05 were considered statistically significant. The predictors of high disability at follow–up were identified through univariate and multivariate analyses. All statistical analyses were performed using SPSS software version 22.

## Results

From January 2003 to May 2023, 95 patients were diagnosed with biopsy-proven PNL.

### Baseline characteristics

The mean age was 43 years (SD 15.32), with a significant male preponderance (77%). Equal proportions of unskilled labourers (35, 36.8%) as well as skilled professionals (37, 38.9%) were affected. Most patients were from North India (64, 67.3%), with a significant proportion from Jharkhand, West Bengal, and Bihar. Diabetes mellitus was seen in 21(22%) patients, and none had HIV infection. Seventy-six (80%) patients presented within 5 years of symptom onset, among whom only 27 (28.5%) presented within 1 year. The demographic data is summarised in Table 1.

**Table 1:** Clinical and electrophysiological characteristics of patients with pure neuritic leprosy.

Characteristics	Value (n = 95)
Age (years, Mean±SD)	42(44.2)
Male sex (%)	76(80)
**Occupation (%)**	
Professional	33(34.7)
Skilled laborers	4(4.2)
Unskilled laborers	31(32.7)
Unemployed	27(28.4)
**Location (%)**	
North India	59(62.1)
South India	26(27.4)
Bangladesh	7(7.3)
Nepal	2(2.1)
Bhutan	1(1.1)
**Comorbidities (%)**	
Diabetes mellitus	21(22.2)
Systemic hypertension	9(9.4)
Human Immunodeficiency Virus Infection	0
Chronic liver disease	2(2.1)
Chronic kidney disease	2(2.1)
**Time to diagnosis from symptom onset (%)**	
Less than 1 year	27(28.5)
1 – 5 years	49(51.5)
More than 5 years	19(20)
**Clinical presentation (%)**	
Sensory deficits	76(80)
Ulnar claw	36(37.8)
Median claw	11(11.1)
Wrist drop	3(3.2)
Foot drop	49(51.5)
Facial palsy	12(12.1)
Trophic ulcers	24(25.2)
Progression to cutaneous lesions	40(42.1)
**Nerve thickening (%)**	
Bilateral involvement	53(55.7)
Supraclavicular	1(1)
Infra-orbital	1(1)
Lateral popliteal	5(5.1)
Ulnar	57(60)
Median	7(7.3)
Radial	23(24.2)
Common peroneal	42(44.2)
Superficial peroneal	7(7.3)
Sural	10(10.5)
Posterior tibial	8(8.4)
Great auricular	6(6.3)
Sciatic	1(1)
**Electrophysiological patterns (%)**	
Mononeuritis multiplex	48(50.5)
Axonal Polyneuropathy	22(23.2)
Demyelinating polyneuropathy	15(15.7)
Mononeuritis	6(6.3)
Plexopathy	4(4.2)
**Management (%)**	
Multi-drug therapy (MDT)	95(100)
Lepra reaction	41(41.4)
Steroids	41(41.4)

### Clinical findings

Focal sensory impairment (76, 80%) was the most common presentation, followed by foot drop (49, 51.5%) and ulnar claw (36, 37.8%). Bilateral involvement (53, 55.7%) was common. Nerve thickening was commonly identified in the ulnar nerve (57, 60%), followed by the common peroneal (42, 44.2%) and radial (23, 24.2%) nerves. Forty patients (42.1%) progressed to develop cutaneous lesions after the onset of nerve paralysis, among which 25(62.5%) patients developed skin lesions within 1 month of initiation of MDT, 3(7%), and 12(30%) after 6 months and 1 year of initiation of MDT. Among these, 17 (42.5%) patients developed skin lesions as part of the lepra reaction type 1.

### Electrophysiological findings

**Fig 1:**
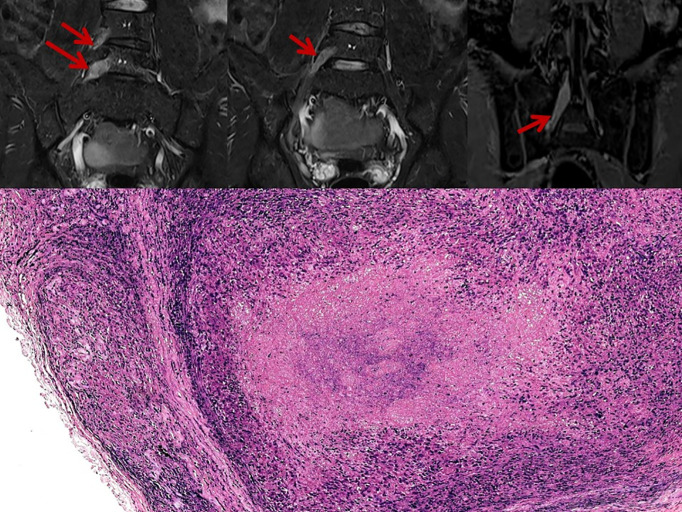
Top panel – Magnetic Resonance Imaging of the Lumbosacral spine showing thickened and enhancing lumbar roots and the sciatic nerve. Bottom panel - Segmental necrotizing granulomatous neuritis evidenced by cross-section of a peripheral nerve expanded by granulomas (HE, 100x) with a central area of necrosis (Arrow). Fite Faraco stain for lepromatous bacilli with occasional acid-fast bacilli. (Fite Faraco stain, 1000x).

The most common electrophysiological pattern was mononeuritis multiplex (48, 50.5%), followed by axonal polyneuropathy (22, 23.2%). Demyelinating polyradiculoneuropathy was seen in 15(15.8%) patients. Four patients (4.2%) presented with atypical symptoms, including lumbosacral plexopathy (Fig 1) or brachial plexopathy, and six patients (6.3%) had mononeuropathy. The clinical and electrophysiological parameters are summarized in Table 1.

### Histopathology

**Fig 2:**
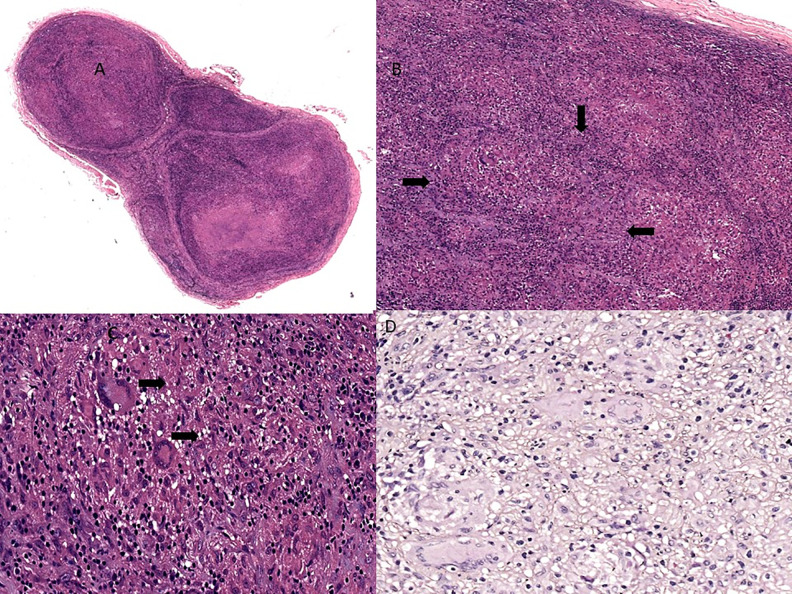
Histopathology showing Borderline Tuberculoid Leprous Neuritis. A - Cross-section of a peripheral nerve expanded by granulomas (HE, 40x). B - Multiple coalescing granulomas infiltrating the nerve (arrows, HE, 200x). C - High power view of granulomas with Langhans-type multinucleated giant cells (arrows, HE, 400x). D- Fite Faraco stain for lepromatous bacilli, which is negative.

**Fig 3:**
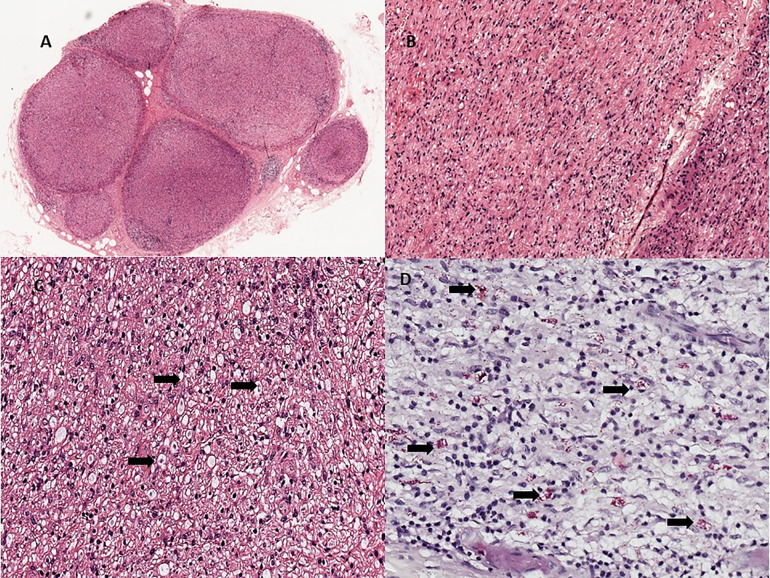
Histopathology of nerve showing Lepromatous neuritis. A - Cross section of a peripheral nerve which is expanded by infiltrates of macrophages and lymphocytes (HE, 40x) B- Longitudinal sections with the inflammatory infiltrates infiltrating the nerve (HE, 200x) C-High power view of foamy macrophages (arrows, HE. 400x) D- Fite Faraco stain showing lepromatous in globi (arrows) and scattered singly (Fite Faraco stain, 400x).

All patients had nerve biopsy confirmed PNL. Seventy-two (75.7%) had borderline tuberculoid (BT) pathology (Fig 2), followed by borderline lepromatous (13.6%). Six patients with mononeuropathy had lepromatous leprosy on nerve biopsy (Fig 3). All the patients with demyelinating polyneuropathy had BT pathology on the nerve biopsy.

### Treatment and Outcomes

**Fig 4:**
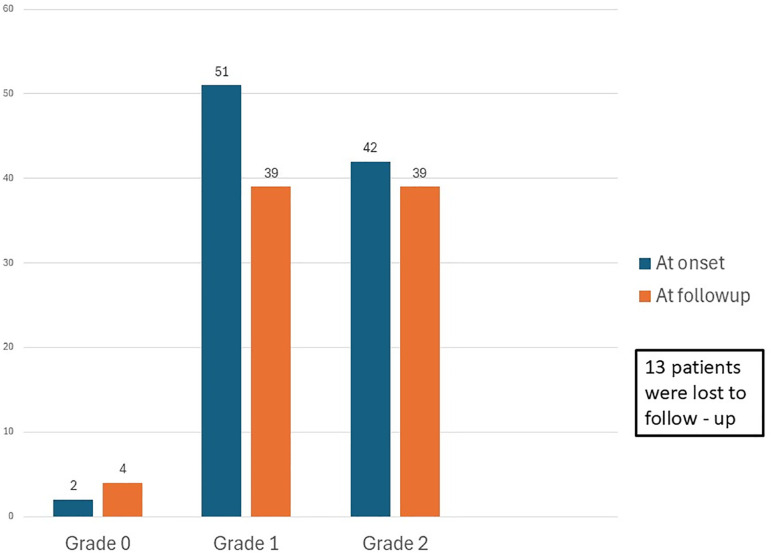
WHO disability grading before and after treatment.

The mean follow-up period was 3 years (IQR: 0.6 – 10 years). All patients received the WHO-standardized MB–MDT regimen. The proportion with high disability at follow-up was 47.5%(n = 39) (Fig 4). Overall, the distribution of disability in the cohort remained relatively stable over time, with 45.3% of patients having a WHO disability grade 2 at onset compared to 47.6% at last follow-up. A total of 11 patients progressed from WHO disability grade 0/1 to grade 2, while 7 patients showed improvement, transitioning from grade 2 to a lower grade. McNemar’s test showed no significant change in disability status between baseline and last follow-up (McNemar statistic = 7, p = 0.48). This stability reflects a balance between patients who experienced worsening and those who demonstrated improvement in disability status. Forty-one patients developed type 1 lepra reactions during treatment.

### Predictors of outcome

In the cohort, 39 patients had poor outcomes. In the univariate analysis, a WHO disability score of 2 at onset and motor impairment were significantly associated with a higher disability at follow-up. Borderline tuberculoid disease (OR 1.68, 95% CI 0.65–3.93; p = 0.45) and lepra reaction (OR 1.60, 95% CI 0.65–3.93; p = 0.42) were not significant predictors.

**Fig 5:**
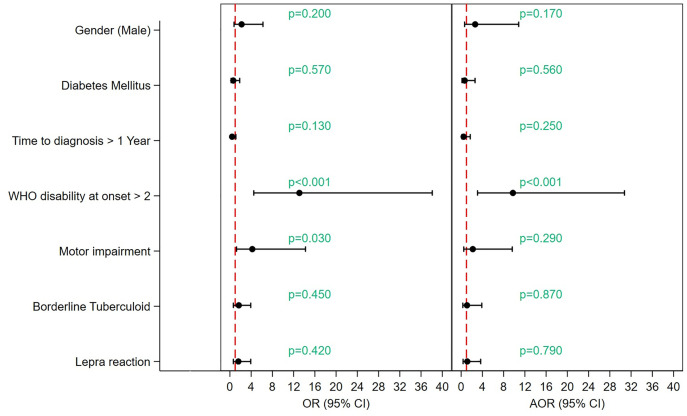
Forest plot showing the predictors of high disability (WHO Grade 2) at follow-up in PNL.

In the multivariate analysis, after adjusting for confounders, only WHO disability score 2 at onset remained independently associated with the outcome (adjusted OR 9.75, 95% CI 3.09–30.75; p < 0.001). Male gender (adjusted OR 2.65; p = 0.17), diabetes mellitus (adjusted OR 0.67; p = 0.56), delayed time to diagnosis (adjusted OR 0.47; p = 0.25), motor nerve involvement (adjusted OR 2.20; p = 0.29), borderline tuberculoid disease (adjusted OR 1.11; p = 0.87), and lepra reaction (adjusted OR 1.16; p = 0.79) were not independently associated with the outcome (Fig 5).

## Discussion

### Summary of Key Findings

In this large, biopsy-confirmed cohort of pure neuritic leprosy (PNL) spanning two decades, we demonstrate that PNL predominantly affects males in the fourth decade and is characterized by substantial diagnostic delay, often extending over several years. This delay likely reflects the insidious onset of subtle sensory deficits, resulting in prolonged, clinically silent nerve injury. Clinically, the ulnar and common peroneal nerves were most frequently involved, with bilateral disease. Nearly half of the cohort presented with advanced disability (WHO grade 2), underscoring the burden of irreversible nerve damage at diagnosis. Electrophysiologically, mononeuritis multiplex was the dominant pattern, consistent with the patchy and asynchronous evolution of nerve involvement. Histopathologically, most cases were paucibacillary with a borderline tuberculoid phenotype and were accompanied by a high incidence of type 1 lepra reactions. Despite the initiation of standard therapy, a substantial proportion of patients experienced persistent or progressive disability, highlighting the limited efficacy of current treatment strategies in preventing long-term neurological sequelae.

### Comparison of prior studies

The demographic characteristics of our cohort are concordant with prior studies, reinforcing the consistent epidemiological profile of PNL [1–3,12,14]. In contrast to earlier reports suggesting a predominance among manual labourers, our cohort demonstrated a more heterogeneous occupational distribution, possibly reflecting evolving socio-demographic patterns or a referral bias inherent to tertiary centres [7,12,15]. The pattern of nerve involvement, predominantly ulnar and common peroneal, and the high frequency of mononeuritis multiplex align with existing literature, supporting the concept of selective vulnerability of superficial nerves subjected to mechanical stress. [2,3,8,15]. Kumar et al., in their report on PNL, identified mononeuritis in 26% of cases and, in 39% of cases, more than one nerve was involved, either unilaterally or bilaterally. [16].

Mononeuritis multiplex was the most common electrophysiological pattern, consistent with the existing literature [6,9,12,17,18]. Segmental demyelination appears to be the pathology, due to the multiplication of organisms within Schwann cells, followed by myelin destruction [2,6,19,20]. Further T cell-dependent cytokine-mediated damage and regeneration lead to axonopathy. [8] Leprosy often causes predominantly axonal neuropathy, which is more severe in the lower limbs. [1,21]. The electrophysiological patterns depend on the timing of the nerve conduction studies; early conduction is often normal, whereas delayed conduction is often associated with conduction blocks and slowed conduction velocities. [22,23]. As the duration increases, axonal damage occurs.

The predominance of the borderline tuberculoid phenotype in our study is consistent with most Indian cohorts, although variability exists across studies regarding the proportion of multibacillary disease. [2,4,6,16,21,24,25]. Kulshrestha et al. demonstrated that single-nerve involvement occurs with multi-bacillary disease (BB, BL, and LL), whereas multiple-nerve involvement indicates a paucibacillary phenotype. [20]. Suneetha et al. reported in their PNL cohort that 46% had multibacillary disease. [21]. A nerve biopsy helps diagnose patients presenting with PNL and guides disease classification, as well as prognosticates disability based on the degree of fibrosis. [20]. In the context of pathogenesis, the BT phenotype has fewer bacilli and a robust cell-mediated immune response. Despite a low antigenic load, extensive tissue damage is the norm in tuberculoid disease due to the host’s inflammatory responses, which contribute to disability. [4,9]. Borderline cases are also prone to develop type 1 lepra reactions when the stable chronic course of the disease is interrupted with antimicrobial therapy.[7,10,12].

### Strengths

This study represents one of the largest single-centre cohorts of biopsy-confirmed pure neuritic leprosy (PNL) reported over a two-decade period. A major strength is the comprehensive integration of clinical, electrophysiological, and histopathological data, enabling robust phenotyping of this neglected disease. The analysis on predictors of disability adds to the existing knowledge on early risk stratification. Importantly, the study highlights a novel, clinically significant observation: patients presenting with mononeuropathy may harbour lepromatous pathology, reinforcing the critical role of nerve biopsy in accurate disease classification and management.

### Limitations

The retrospective design of the study inherently limits causal inference and introduces the possibility of missing data, documentation bias, and unmeasured confounding. As the study was conducted at a single tertiary referral centre, there is a risk of referral bias, with a higher likelihood of inclusion of more severe or atypical cases, thereby limiting generalizability to community or primary care settings. Additionally, including only biopsy-confirmed cases may have excluded milder or clinically diagnosed PNL, potentially underrepresenting the full disease spectrum. Heterogeneity in follow-up duration across patients may compromise consistency in outcome assessment.

## Conclusions

In endemic countries, PNL appears to be a significant problem and must be kept in the differential diagnosis when evaluating a patient with peripheral neuropathy, as early diagnosis and initiation of multi-drug therapy is prudent to halt further nerve damage. A nerve biopsy is necessary to accurately classify the disease. A higher disability at onset appears to be an important predictor of persistent disability at follow-up. Molecular methods and host susceptibility factors should be systematically studied to facilitate improved diagnostics, identify more effective antimicrobial drugs, and efficiently manage host immune responses.
